# Genetic Modulation at the Neural Microelectrode Interface: Methods and Applications

**DOI:** 10.3390/mi9100476

**Published:** 2018-09-20

**Authors:** Bailey M. Winter, Samuel R. Daniels, Joseph W. Salatino, Erin K. Purcell

**Affiliations:** 1 Department of Biomedical Engineering, Michigan State University, East Lansing, MI 48824, USA; winterb8@msu.edu (B.M.W.); danie276@msu.edu (S.R.D.); joeysal@msu.edu (J.W.S.); 2 Department of Electrical and Computer Engineering, Michigan State University, East Lansing, MI 48824, USA

**Keywords:** microfluidic device, chronic implantation, gene modification

## Abstract

The use of implanted microelectrode arrays (MEAs), in the brain, has enabled a greater understanding of neural function, and new treatments for neurodegenerative diseases and psychiatric disorders. Glial encapsulation of the device and the loss of neurons at the device-tissue interface are widely believed to reduce recording quality and limit the functional device-lifetime. The integration of microfluidic channels within MEAs enables the perturbation of the cellular pathways, through defined vector delivery. This provides new approaches to shed light on the underlying mechanisms of the reactive response and its contribution to device performance. In chronic settings, however, tissue ingrowth and biofouling can obstruct or damage the channel, preventing vector delivery. In this study, we describe methods of delivering vectors through chronically implanted, single-shank, “Michigan”-style microfluidic devices, 1–3 weeks, post-implantation. We explored and validated three different approaches for modifying gene expression at the device-tissue interface: viral-mediated overexpression, siRNA-enabled knockdown, and cre-dependent conditional expression. We observed a successful delivery of the vectors along the length of the MEA, where the observed expression varied, depending on the depth of the injury. The methods described are intended to enable vector delivery through microfluidic devices for a variety of potential applications; likewise, future design considerations are suggested for further improvements on the approach.

## 1. Introduction

An increasing prevalence of patients affected by neurodegenerative diseases and psychiatric disorders places an economic, social, and psychological burden on society [1,2,3,4,5,6]. In research settings, there has been a significant rise in the use of microelectrodes implanted in the brain to record neural activity and reveal the underlying mechanisms of these diseases. Moreover, brain-machine interfaces (BMIs) and closed-loop deep brain stimulation (DBS) are emerging applications of recording arrays, in preclinical and clinical trials [7,8,9,10,11]. However, their signal qualities are notoriously unstable and are prone to loss over time, which undermines the efficacy of decoding algorithms, the accuracy of data collection in basic science studies, and the detection of conditioning signals necessary to drive the closed-loop strategies [12,13,14,15]. The brain initiates a tissue response, following implantation, that is characterized by a progressive glial encapsulation and neuronal loss, which is widely believed to contribute to the diminished recording quality and signal loss [14,16,17]. However, despite recent findings, both, the nature of the relationship between the tissue response and recording quality, as well as the underlying mechanisms responsible, remain unclear [18].

In recent years, an increasingly complex view, of tissue response to neural implants, has emerged, where changes in the structure and function of the responding cell-types accompany well-known effects on cellular density (glial encapsulation and local loss of neurons). Recent evidence suggests that the local shifts in ion channel expression, synaptic transporter expression, and astrocyte subtype follow from a device implantation [14,19]. Additionally, Eles et al. noted new evidence that mechanical trauma accompanies prolonged, localized calcium influx, post-implantation [20]. For non-neuronal responses, a recent study employed a mouse bone marrow chimera model as an innovative approach to delineate the roles of resident microglia versus blood-derived macrophages, in determining the microelectrode performance. The study revealed that the knockout of CD14 from blood-derived macrophages improved the recording quality, over 16 weeks [21]. Further, new approaches using extracellular matrix-based intracortical arrays have reduced inflammatory responses, demonstrating the important role of acellular elements in modulating the tissue response [22,23]. While these findings provide insight on the fundamental mechanisms of the tissue response, they also illustrate the complexity of the device-tissue interactions and the significant unknowns that remain, with respect to the signaling pathways responsible.

Advances in the development of new genetic tools provide opportunities to identify the precise pathways of cellular responses, where devices are being designed and fabricated with increasingly sophisticated means of delivering the necessary reagents to the surrounding tissue. Multifunctional microelectrode arrays offer the ability to interrogate the cellular events surrounding the device, via electrical, chemical, and optical modes of stimulation [24,25,26,27], and integrated microfluidic channels permit the vector delivery for genetic modification of the local neural network [27,28]. The resulting upregulation or downregulation, of the specific signaling pathways, is a potentially powerful means of investigating the mechanisms of the tissue response. However, difficulties can arise in chronic settings, since biofouling and tissue ingrowth can compromise the patency of the infusion channel, making repeated dosing, and vector delivery at long-term time points, challenging [27,29,30].

Here, we present data illustrating a proof-of-principle for delivering vectors capable of modifying gene expressions (siRNA and viral vectors), via a functional microfluidic device capable of recording neural activity in the primary motor cortex of adult rats. By delivering reagents designed for gene knockdowns (BLOCK-iT^TM^ siRNA, Thermo Fischer, Waltham, MA, USA), overexpressions (AAV8-GFAP-mCherry, UNC Vector Core, Chapel Hill, NC, USA), and conditional expressions (AAV2-Cre-GFP, Vector Biolabs, Philadelphia, PA, USA), our results provide a methodology for the genetic modification, of the tissue response, at the neural-electrode interface.

## 2. Materials and Methods

### 2.1. Injection Protocol (In Vitro)

**Figure 1:**
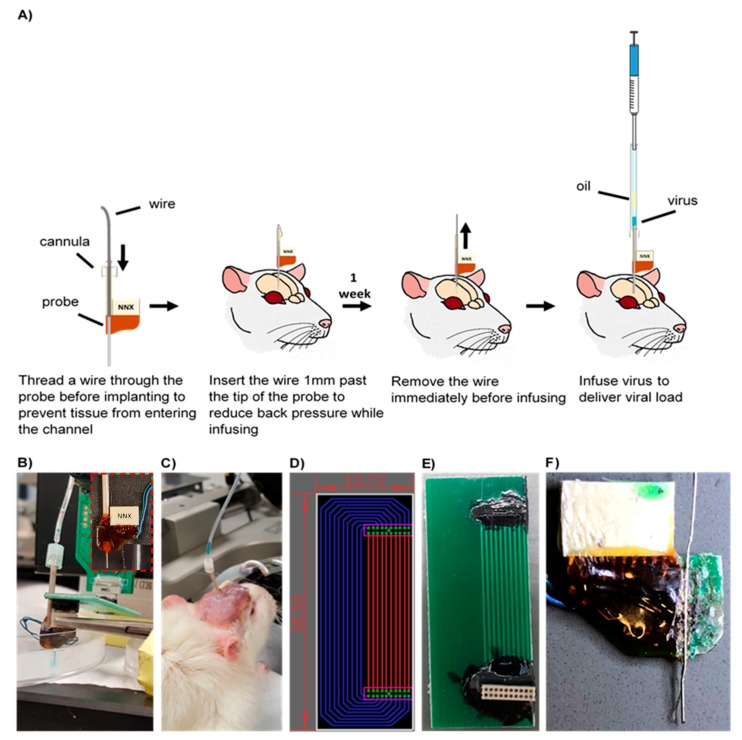
(**A**) Microfluidic device implantation and infusion protocol. (**B**) In vitro saline infusion into 0.6% agarose. Saline was tinted with fast green to confirm delivery. Inset displays the microfluidic device. (**C**) In vivo infusion of the AAV8 viral load. (**D**) Top view of the adapter board layout. Green circles indicate the plated through-holes through which the electrical connector (Omnetics, Minneapolis, MN, USA) leads were inserted. Electrical traces were placed on the top (red) and bottom (blue) to prevent traces from overlapping. Dimensions indicated in the figure are in millimeters. (**E**) Fabricated adapter board. (**F**) Cross section of a microfluidic probe. Red arrow indicates a slight bend in the microfluidic channel.

The workflow of the injection protocol developed is illustrated in Figure 1A. A custom 16-channel single shank microfluidic microelectrode array (Neuronexus, Ann Arbor, MI, USA) was pre-threaded with a 40 gauge SS316L wire (KidneyPuncher, Mesa, AZ, USA). Due to the slight bend in the microfluidic channel, near the electrical connector (Figure 1F), an infusion cannula (33 gauge internal cannula, C315LI/SPC PlasticsOne, Roanoke, VA, USA) was used as a guide for the wire insert. For initial in vitro testing and methods development, the MEA was inserted into a brain tissue phantom, consisting of a 0.6% agarose hydrogel, cast into a 10 cm cell culture petri dish, and was held in place using a hemostat and C-clamps (Figure 1B,C) [31]. Subsequently, the wire was inserted 1 mm past the tip of the MEA into the agarose medium, to clear the microfluidic channel and reduce the back pressure. To infuse, the infusion cannula was first attached to a 10 uL Hamilton syringe, with 7 cm silicon tubing (C313CT, PlasticsOne, Roanoke, VA, USA). Using a Quintessential Stereotaxic Injector (Stoelting, Gelsenkirchen, Germany), 4 μL of mineral oil was withdrawn at 0.1 μL/min, followed by 2 μL of air, and finally, 2 μL of saline, tinted with fast green (Electron Microscopy Sciences, Hatflied, PA, USA), at the same rate. Using hemostats, the cannula was carefully inserted into the microfluidic channel and glued in place. The saline was then infused into the agarose medium at a rate of 0.1 μL/min (a standard infusion rate for vector injection into brain tissue) [32].

### 2.2. Adapter Circuit

Due to the close proximity of the microfluidic channel, an adapter circuit was designed and fabricated to allow added clearance between the connector and the infusion channel, and facilitate the collection of neural recording data. Using the EAGLE schematic software (Autodesk, San Rafael, CA, USA), the circuit board was designed to extend the connection site 1 inch away from the device (fabricated by Gold Phoenix PCB, Wuhan, China) (Figure 1D,E). Electrical connectors (Omnetics, Minneapolis, MN, USA) with through-hole style leads were chosen for ease of assembly, and connection pads were determined based on manufacturer specifications.

### 2.3. Surgical Procedure

For the conditional expression, we purchased a reporter rat strain from the National BioResource Project (Kyoto University, Kyoto, Japan) which enables high-resolution imaging of dendritic spines in ex vivo brain slices [33]. For the reporter strain, female Long Evans rats were generated with a floxed STOP tdTomato to allow conditional expression of the red fluorescent reporter, via viral delivery of Cre recombinase (4.0E12 GC/mL AAV2-Cre-GFP in 5% Glycerol in PBS, Vector Biolabs, Philadelphia, PA, USA). For overexpression and knockdown studies, adult male Sprague–Dawley rats (Charles River, Wilmington, MA, USA) were delivered an AAV vector, with a GFAP promoter and mCherry reporter (AAV8-GFAP-mCherry 2.7E12 virus molecules/mL in 350 nM NaCl and 5% D-Sorbitol in PBS, UNC Vector Core, Chapel Hill, NC, USA) and BLOCK-iT^TM^ siRNA (200 nM Invivofectamine-BLOCK-iT^TM^-Complexation solution, Thermo Fisher, Waltham, MA, USA), respectively. Animals were unilaterally implanted in the motor cortex, using a commercially manufactured 16-channel single shank MEA, with a microfluidic channel (NeuroNexus, Ann Arbor, MI, USA) that was pre-threaded with a 40 gauge SS316L wire (KidneyPuncher, Mesa, AZ, USA), based on previously published methods that used single shank standard (non-microfluidic) Michigan arrays [19]. The majority of the implanted microfluidic devices were nonfunctional. All devices were modified so that the plastic tubing was ~2 cm long. Animals were anesthetized with ~2% isoflurane, throughout the surgery. Using a motorized drill, a 2 mm × 2 mm craniotomy was performed to expose the cortex (3 mm anterior, 2.5 mm lateral to bregma). The dura was resected and the MEA was implanted at a 2 mm depth, in the cortex. Subsequently, the wire was manually threaded and inserted ~1 mm past the tip of the MEA. A dental acrylic headcap, anchored by three bones screws, was used to support the MEA. Excess dental acrylic was used to attach the wire to the plastic tubing of the microfluidic channel. Bupivacaine was administered for topical analgesia, at the wound site, and meloxicam was administered for systemic analgesia, via an intraperitoneal injection during recovery. All surgical procedures were approved by the Michigan State University Animal Care and Use Committee (Project identification code AUF # 11/17-196-00).

### 2.4. Injection Protocol (In Vivo)

Animals were infused 1–3 weeks, post-implantation. Animals were anesthetized with ~2% isoflurane for the duration of the procedure. Oil, air and 2 μL of viral load were withdrawn, as described in the Injection Protocol section. The wire was removed from the microfluidic channel and the cannula was inserted, using hemostats, and was glued in place. The viral load was infused at 0.2–0.4 μL/min. For troubleshooting techniques, see Box 1.

Box 1 **Troubleshooting techniques for in vivo infusions.**

**Troubleshooting Techniques**

**The channel became clogged**

If the wire insert was pulled out, tissue could infiltrate and clog the microfluidic channel, preventing a successful infusion. In order to clear the channel, a new wire must be inserted; however, a blunt wire might not be able to pierce through the debris. Filing the tip of the wire to a point could help clear the debris from the channel and might require multiple attempts.

**The plastic tubing broke off**

The plastic tubing could be damaged or completely broken through contact with the environment, as the animal explored its cage. If the plastic tubing could be recovered from the enclosure, it could be reattached with super glue, immediately before infusion. If the tubing could not be recovered, a guide cannula (PlasticsOne, Roanoke, VA, USA) could be used as a substitute.

**The microfluidic channel became damaged**

If the plastic tubing was broken, the microfluidic channel could become damaged or could be removed, as well. If a portion of the microfluidic channel was still attached, the channel could be straightened out and reattached, or the plastic tubing could be replaced. If the microfluidic channel had broken off completely, the channel had to be reconstructed. First, it had to be ensured that the microfluidic channel was clear of debris. A cannula could be threaded with a wire insert, and inserted into the plastic tubing/guide cannula, until the ends were aligned. The wire should be extended far enough, past the opening, so that it could be easily manipulated. Using forceps, the wire could be guided into the microfluidic channel to align the cannula and plastic tubing/guide cannula. Super glue could be applied to the bottom of the plastic tubing/guide cannula to allow it to set. Finally, the cannula and wire insert could be removed.

**The virus did not infuse**

If the virus did not infuse, it was possible that the wire was not inserted far enough and the channel was blocked with tissue. A wire could be re-inserted through the channel until it extended approximately one millimeter past the end of the microfluidic channel. If the virus still did not infuse, the infusion rate was increased slightly, until successful.

### 2.5. Immunohistochemistry

Two to three weeks post-injection, animals were deeply anesthetized with an overdose of sodium pentobarbital, and transcardially perfused with 4% paraformaldehyde (PFA). Brains were extracted, stored in 4% PFA overnight and cryoembedded, following sucrose protection. Cryosections were collected at a 20 μm thickness and hydrated with PBS, prior to blocking in a 10% normal goat serum in PBS for 1 h. Tissue was subsequently incubated overnight, at 4 °C, with the mouse anti-glial fibrillary acidic protein (GFAP) (Cell Signaling Technology, Danvers, MA, USA). The following day, cryosections were rinsed with PBS and incubated with the goat anti-mouse IgG (H+L) Alexa Fluor 488 conjugate (1:200, Thermo Fisher Scientific, Waltham, MA, USA), for two hours, at room temperature. Finally, nuclei were counterstained with Hoechst and coverslipped with ProLong Gold antifade reagent (Fisher Scientific Company, Hampton, NH, USA). An Olympus Fluoview 1000 inverted confocal microscope was used to image samples with a 20× PlanFluor dry objective (0.5NA). For comparison, GFAP-stained tissue from “traditional” (non-microfluidic) single shank Michigan-style arrays, implanted in the motor cortex, was assessed using images collected during a previous study [34]. 

### 2.6. Image Analysis

All images were analyzed using a MATLAB script adapted from Kozai et al. [15] with modifications previously reported [19]. A hand-traced outline of the injury was used to define concentric 10 μm-thick bins. The average intensity of the fluorescent markers within each bin was calculated using the corners of the image as a reference. Bin intensity was normalized to the most distal bin. Results were assessed using a mixed model ANOVA and SPSS software (IBM, Chicago, IL, USA) as previously described [19].

### 2.7. Signal Processing

Neural recording data were acquired with a Tucker-Davis Technologies RZ2 system (Alchua, FL, USA) and processed using a MATLAB script. Wideband data was sampled at ~48 kHz, in isoflurane-anesthetized rats placed in a Faraday cage, and analyzed offline as described [34,35]. A combination of bandpass filtering and identification of threshold crossings (at 3.5 standard deviations from the mean of the sampling distribution) were used to collect and store 3 ms snippets, centered at the minimum of the recorded segment. Local field potentials (LFPs) were filtered between 1–100 Hz. LFP amplitude was calculated by multiplying the standard deviation of the signal by six, yielding 99.7% of the signal amplitude. Principal component analysis and fuzzy C-means clustering (membership index > 0.8) were performed to identify putative units, in combination with visual inspection of the mean waveforms. Common average referencing [36] was used to mitigate noise sources common to every electrode site (such as line noise and movement artifacts).

## 3. Results

### 3.1. Development of Injection Methods (In Vitro)

Optimal infusion methods were determined through trial and error in vitro, prior to implementation in vivo. The rate of withdrawal or infusion of each component (oil, air, and virus) was found to be a critical determinant of success for the overall procedure. When withdrawn at a rate higher than the optimum (0.1 μL/min), bubbles would form within the oil, preventing a successful infusion. Additionally, due to the viscosity of the oil, higher rates of withdrawal resulted in inaccuracies in the volume of oil collected. Subsequently, if the air was withdrawn at a higher rate, bubbles would form in the oil. Likewise, withdrawing the viral load at higher rates could result in withdrawal of air into the sample and an unsuccessful infusion.

### 3.2. Increased GFAP Expression Surrounding Microfluidic Devices

**Figure 2:**
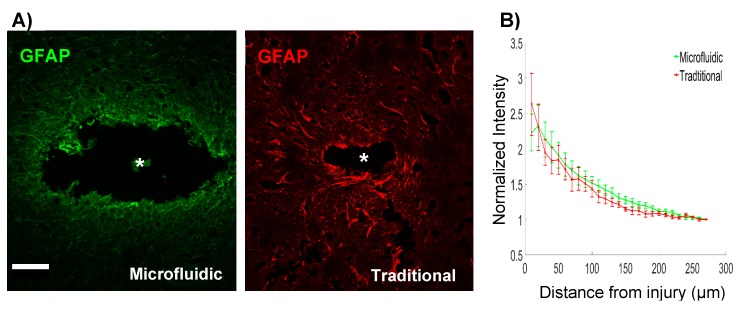
(**A**) GFAP staining surrounding microfluidic and traditional devices indicates astrogliosis and a larger injury footprint related to the microfluidic device. Scale bar = 100 μm. (**B**) Microfluidic devices show significantly elevated levels of GFAP expression within 130 μm of the injury, in comparison to the distal control values (*p* < 0.05). Traditional devices have a slightly more compact region of gliosis, with significantly elevated levels of GFAP within 100 μm (*p* < 0.05). * denotes injury center.

The inclusion of a microfluidic channel on the device necessarily increased the footprint of the implant. Given the evidence for a relationship between device architecture and tissue response [37,38], we explored the level of astrogliosis surrounding the microfluidic devices, in comparison to “traditional” single shank, silicon probes. Microfluidic devices created larger injuries compared to traditional devices, with average injury areas of 0.056 mm^2^ and 0.004 mm^2^, respectively (Figure 2A). Although the width of the microfluidic device was 185 μm, injury sizes were noticeably larger. Hoechst staining confirmed the absence of cells within the injury area (not shown). This exacerbated injury size could be due to the removal of the tissue that adhered to the device while extracting the brain. This larger injury size was accompanied by increased astrogliosis. Quantification of GFAP fluorescence surrounding microfluidic devices showed significantly elevated levels of GFAP expression (*p* < 0.05), up to 130 μm of the insertion site boundary (relative to the distal bin intensity values). Traditional devices showed a slightly more spatially restricted response, with significantly elevated levels of GFAP expression (*p* < 0.05) detected up to 100 μm from the device tract. Furthermore, a trend toward an overall elevation in GFAP expression was detected in microfluidic devices, in comparison to the traditional devices (*p* < 0.1) (Figure 2B). These results indicated that the larger footprint of the microfluidic device could slightly exacerbate the reactive astrogliosis. 

### 3.3. Signal Quality of Microfluidic Devices

**Figure 3:**
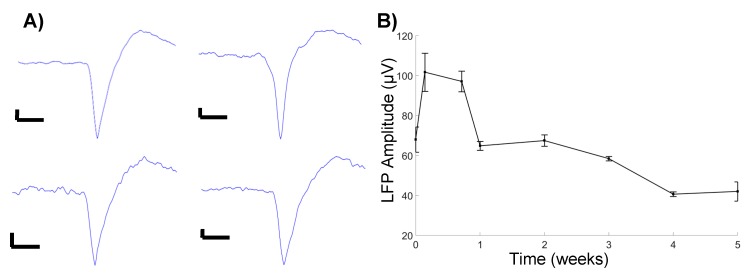
(**A**) Recorded units. All scale bars are 10 μV amplitude, 0.5 ms timescale. (**B**) Average LFP amplitude.

We observed an initial increase in LFP amplitude one day, post-implantation, followed by a gradual decrease in the amplitude, before stabilizing at four weeks, post-implantation (Figure 3B). These observations follow a general trend of decreasing signal quality over time. Additionally, a cursory observation yielded limited identification of unit activity, with the units being detected only at 5 days, post-implantation (Figure 3A). While the sample size was limited, the observation confirms the ability to successfully detect unit activity with microfluidic devices, in a chronic setting.

### 3.4. Microfluidic Devices Successfully Deliver Virus along the Length of the Injury

Animals were infused 1–3 weeks, post-implantation according to the methods developed in vitro. Some alterations to the infusion protocol developed in vitro were necessary to successfully deliver the viral load in vivo. An infusion rate of 0.1 μL/min proved insufficient to overcome the back pressure in an in vivo setting. Increasing the infusion rate to 0.2 μL/min was sufficient for most animals; however, variations between animals required infusion rates up to 0.4 μL/min for successful virus delivery.

**Figure 4:**
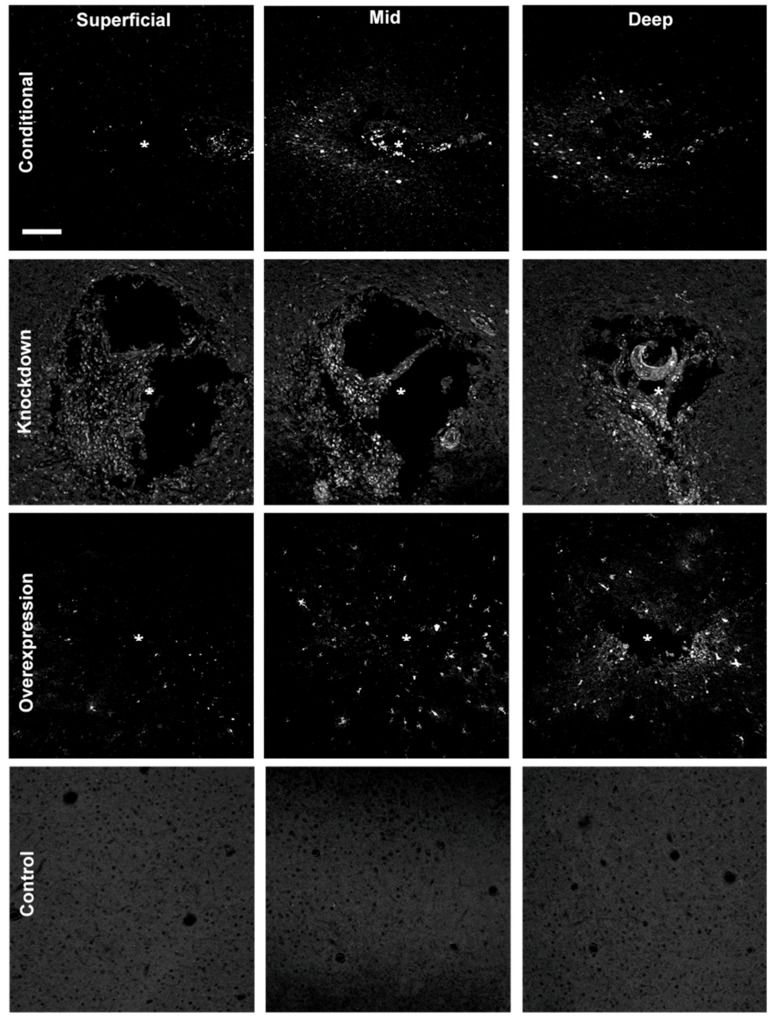
Spread of fluorescent reporter expression (appears white) of AAV8-GFAP-mCherry (overexpression), BLOCK-iT^TM^ siRNA (knockdown), and AAV2-Cre-GFP (conditional) at superficial (~100–650 μm), mid (~750–1000 μm), and deep (~1100–1300 μm) sections of the injury. Reporter expression is spatially broader in deep sections of the injury (near the infusion tip) in comparison to more superficial sections. Control images were taken from the contralateral hemisphere; * denotes injury center. Scale bar = 100 μm.

Animals were sacrificed 2–3 weeks after infusing, and cryosections were imaged with an Olympus Fluoview 1000 confocal microscope to assess the expression of the infused virus. All successfully infused animals showed expression of the delivered fluorescent reporter surrounding the injury site (Figure 4). We observed a qualitative increase in the amount of expression, and diffusion of the vector at deeper sections of the injury, compared to the superficial sections. This pattern of dispersion is most likely due to the channel having a single opening at the tip of the probe.

## 4. Discussion

While it is widely believed that the biological response to implanted electrodes is intimately linked to their function [16,17], direct evidence for this relationship is surprisingly scarce. Multiple studies have explored correlations between a specific measure of recording quality (such as the number of units detected), impedance data, and/or an isolated metric of the biological response (for instance, local neuronal density) to gain insight into the association between functional characteristics and cellular responses. However, the variability in the outcomes reported in these studies underscores the complexity and multi-faceted nature of the underlying source(s) of recorded signal loss, interface instability, and shifting stimulation thresholds [18,39,40]. Likewise, while new device designs incorporate increasingly sophisticated features and materials [41], the relationship between each of these design features, the impacts on tissue response, and chronic device performance remain poorly characterized. The development of new tools and test beds, to understand the basic science governing tissue-device interactions, could provide a direct link between biological mechanisms and device function, ultimately delivering guiding principles for the design features necessary to enable improved tissue integration.

In the current study, we developed and validated methods to modify gene expression using multiple techniques (silencing, conditional expression, and overexpression). Each of these approaches provides a new “knob” to turn, to tune the biological response to devices in controlled ways, potentially providing a mechanistic link between observations of localized changes in gene and protein expression and device performance. This approach builds on previous work that has used drug-based strategies to modulate the biological response to electrodes, either through exacerbation or mitigation of effects, to explore the role of neuroinflammation in signal loss. For example, microelectrodes in animals administered lipopolysaccharide, a common pro-inflammatory stimulus, had a notably lower signal-to-noise ratio and fewer units detected, as compared to the control rats; whereas, anti-inflammatory drugs have been shown to decrease neuroinflammation and improve recording quality [17,42,43,44,45]. However, targeting specific signaling pathways, through a perturbation of the gene expression, may offer a more granular view of the key biological mechanisms mediating the neurotoxicity and inflammation that occur at the neural-electrode interface.

As more information on the genes differentially expressed at the device interface becomes available, new opportunities to identify relevant candidate pathways will emerge. Recent studies evaluating gene expression surrounding implanted devices noted an increased expression of GFAP, TNFα, NOS2, HMGB1, CD14, and numerous members of the IL gene family [46,47,48]. Additionally, Bennett et al. showed genes regulating tight junction and adherens junction proteins in the blood-brain barrier were downregulated, after 72 h, post-implantation [48]. Manipulation of gene expression, by upregulation or downregulation, could reveal potential breakthroughs in improving device function and integration in the brain. Further, Cre-recombinase allows an added layer of control of the local gene expression, by enabling the conditional knockout and genome editing [49].

While the approaches developed in this study were successful in the localized modulation of gene expression, several areas for future improvements were identified. The epoxy used to attach the plastic tubing to the probe was brittle and prone to cracking. This resulted in the potential loss of the plastic tubing during the natural exploratory behavior of the subject, ultimately leading to the microfluidic channel becoming damaged. A more flexible epoxy could reduce the likelihood of this issue. Additionally, the proximity of the microfluidic channel to the electrical connector necessitated the use of an adapter, in order to avoid damaging the channel, while recording the neural activity. While not affecting our ability to infuse, having a single opening at the tip of the probe resulted in an unequal distribution of the delivered virus, along the length of the probe. Alternative designs that enable more control over the distribution and location of viral delivery would be beneficial for applications that seek to perturb the environment, at specified sites, along the length of the probe.

Recent efforts in neural engineering have accentuated the need for understanding the basic science behind the biological mechanisms at the device interface. Next-generation device design is contingent on identifying these unknown device-tissue interactions. This work provides an approach to interrogate and understand the local environment around an implanted device, enabling new opportunities to investigate the tissue response to implants, and identify improved device designs.
